# The short-term effect of vitamin D supplementation on the response to muscle and liver damages indices by exhaustive aerobic exercise in untrained men: a quasi-experimental study

**DOI:** 10.1186/s13102-022-00398-1

**Published:** 2022-01-10

**Authors:** Vahid Parvizi Mastali, Rastegar Hoseini, Mohammad Azizi

**Affiliations:** Department of Exercise Physiology, Faculty of Sport Sciences, Razi University, No. 9, Taq Bostan, University Street, P.O. Box, 6714414971 Kermanshah, Iran

**Keywords:** Exhaustive aerobic exercise, Overweight, Vitamin D, Muscle and liver damage indices

## Abstract

**Background:**

Exercise-induced muscle damage typically caused by unaccustomed exercise results in pain, soreness, inflammation, and muscle and liver damages. Antioxidant supplementation might be a useful approach to reduce myocytes and hepatocytes damages. Therefore, the present study was conducted to investigate the effect of short-term vitamin D (Vit D) supplementation on the response to muscle and liver damages indices by Exhaustive Aerobic Exercise (EAE) in untrained men.

**Methods:**

In this clinical trial, 24 untrained men were randomly divided into experimental (Exp; n = 12) and control (C; n = 12) groups. Exp received 2000 IU of Vit D daily for six weeks (42 days), while C daily received a lactose placebo with the same color, shape, and warmth percentage. Two bouts of EAE were performed on a treadmill before and after six weeks of supplementation. Anthropometric characteristics (Bodyweight (BW), height, Body Fat Percentage (BFP), Body Mass Index (BMI), waist to hip ratio (WHR)) were measured at the Pre 1 and Pre 2. Blood samples were taken to measure the Creatine Kinase (CK), Lactate Dehydrogenase (LDH), Aspartate Aminotransferase (AST), Alanine Aminotransferase (ALT), Gamma-Glutamyl Transferase (GGT), Alkaline Phosphatase (ALP), and Vit D levels at four stages: Pre 1 (before the first EE session), Post 1 (after the first EE session), Pre 2 (before the second EE session), and Post 2 (after the second EE session). The data were analyzed using repeated-measures ANOVA, Bonferroni's post hoc test, independent *t* test, and dependent t-test at the significant level of *P* < 0.05 using SPSS version 26.

**Results:**

The results show significant differences between Exp and C in alterations of BW (*P* = 0.039), BMI (*P* = 0.025), BFP (*P* = 0.043), and WHR (*P* = 0.035). The results showed that EAE increased muscle and liver damage indices and Vit D (*P* < 0.05). Compared with C, the results of the independent t-test showed significantly lower ALT (*P* = 0.001; *P* = 0.001), AST (*P* = 0.011; *P* = 0.001), GGT (*P* = 0.018; *P* = 0.001), and ALP (*P* = 0.001; *P* = 0.001); while significantly higher Vit D (*P* = 0.001, *P* = 0.001) in the Exp in both Pre 2 and Post 2; receptivity. The independent *t* test showed significantly lower ALT (*P* = 0.001; *P* = 0.001), AST (*P* = 0.011; *P* = 0.001), GGT (*P* = 0.018; *P* = 0.001), and ALP (*P* = 0.001; *P* = 0.001) and considerably greater Vit D (*P* = 0.001, *P* = 0.001) in the Exp in both Pre 2 and Post 2 compared to C. The results of an independent *t* test showed that LDH and CK levels in the Exp were significantly lower than those in the Post 2 (*P* = 0.001).

**Conclusions:**

Short-term Vit D supplementation could prevent myocytes and hepatocytes damage induced by EAE.

## Background

Being overweight is one of the common health problems that cause chronic severe conditions. Exercise training and a healthy diet are non-pharmacological approaches to losing weight [1, 2]. According to the studies, hepatic and skeletal muscle cells damage is observed at the beginning of an exercise program, especially in previously untrained individuals (due to the large volume of unaccustomed contractions) [3, 4]. Changes in some metabolic and mechanical factors following Exhaustive Aerobic Exercise (EAE) may cause direct or indirect damage to the cell membrane, infiltration of intracellular components into the extracellular fluid leading to hepatocytes and myocytes damage consequently [5, 6]. EAE leads to the infiltration of Creatine Kinase (CK) and other intracellular proteins (e.g., Lactate Dehydrogenase (LDH) enzyme) to the interstitial fluid, which is infused into the bloodstream after being collected by the lymphatic system [7]. The results of the current literature show increased skeletal muscle cells damage indicators following acute EAE [8, 9]. Various methods have been proposed as potential therapeutic options to reduce hepatic and skeletal muscle cell damage induced by EAE, including antioxidants, and dietary supplements [10, 11]. Vitamin D is one of the fat-soluble vitamins with antioxidant and anti-inflammatory characteristics that have recently been a subject of debate [12]. It has been hypothesized that vitamin D deficiency could play a critical role in the pathogenesis and progress of many neuromuscular diseases, suggesting Vit D as a useful strategy to enhance muscle function in different populations [13]. Optimal vitamin D levels suppress inflammatory reactions by helping to maintain adequate levels of appropriate anti-inflammatory cytokines (mainly TNF-α and interleukin-10) [14]. In addition, by activating intracellular receptors, vitamin D increases the protein content of cells, increases muscle strength and endurance (maintaining proper ATP levels) [15, 16], protects muscle fibers degeneration [17], and delays the onset of muscle pain and fatigue [18].

Since hepatic and skeletal muscle cells damage in untrained overweighed individuals lowers the adherence to the exercise program and because of the efficacy of anti-inflammatory and antioxidant properties of vitamin D supplementation in preventing EAE-induced damage and weight control, this study aimed at investigating the effect of short-term vitamin D supplementation on the response of hepatic and skeletal muscle cells damage indicators following EAE in untrained men.

## Method

### Study design and participants

The trial (IR.RAZI.REC.1400.001) was approved by the Ethics Committee of the Kermanshah Razi University and registered in the Iranian Clinical Trial Registration Center under the code IRCT20210414050965N1. Written informed consent was obtained from all participants, including the patients’ agreement to participate as volunteers and the possibility to withdraw from the study.

**Fig. 1:**
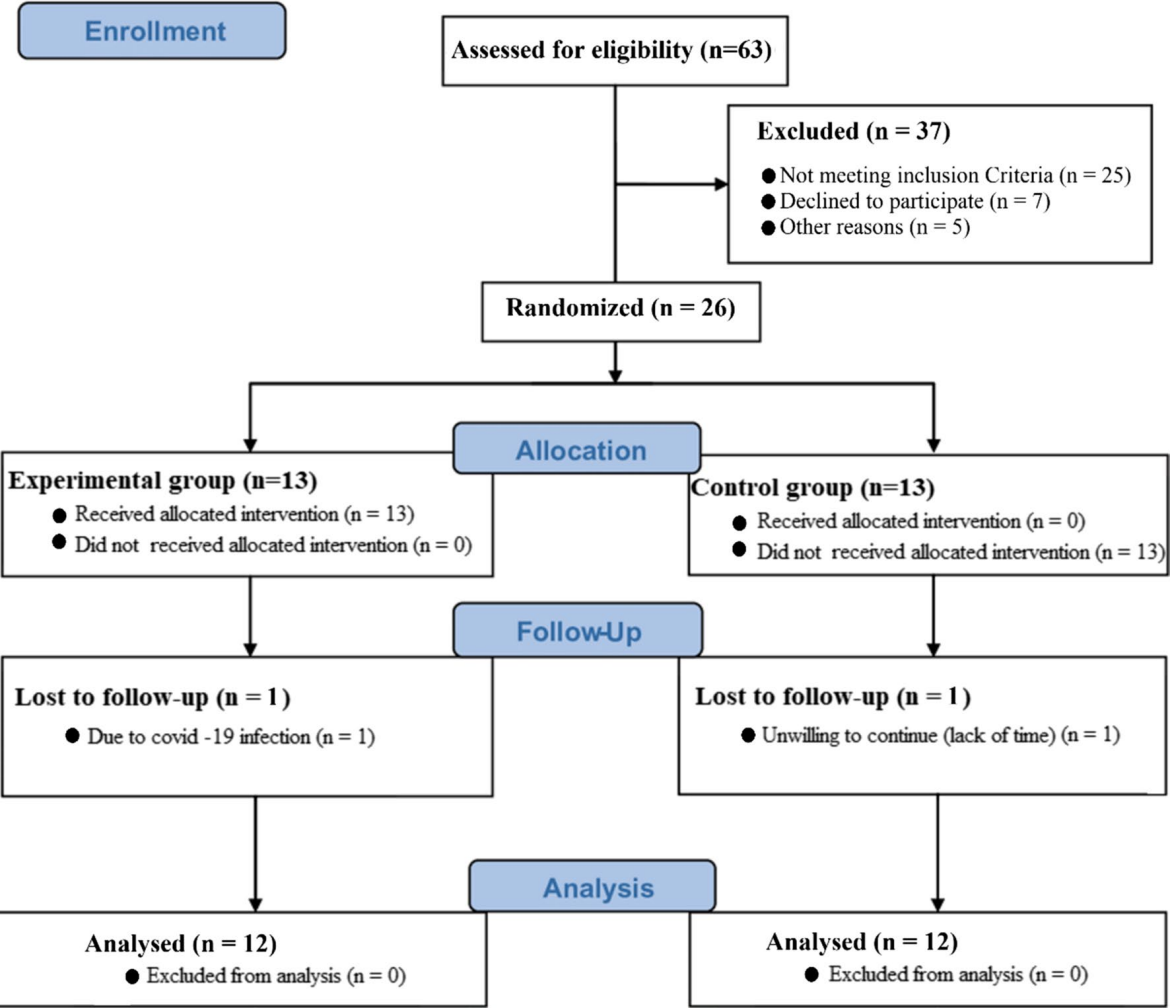
Consort flow diagram for the study

This is a quasi-experimental study with pre-test and post-test design with the placebo group. The statistical population of this study was non-athlete men (aged 20–30 years, skin phototypes from II to IV, Kurd ethnicity, manly students or graduated, with a regular sleeping pattern, with limited sun exposure due to clothing and lifestyle) living in Kermanshah. Invitations for participating in the clinical trials were posted on Telegram, Instagram, and WhatsApp to select the subjects. Forty-eight individuals volunteered, among which 26 who met the inclusion criteria were selected as subjects. Inclusion criteria included: Vit D deficiency (25-hydroxyvitamin D below 30 ng/mL), lack of underlying disease, musculoskeletal injury, regular physical activity in the last six months and during the intervention, infection with COVID-19, any kind of supplementation, and smoking. One day before the start of the study, the subjects were given the necessary explanations about the research procedures and signed the informed consent of participation in the research. Also, the three-day estimated food record, nutrition (including dietary history, food frequency, eating habits, and behaviors), and health questionnaires were filled. Health questionnaires included: history of previous diseases (hypertension, asthma, vascular problem), family history of diseases, medical history, assessment of physical activity level (activity level, duration of activity). Anthropometric characteristics (Bodyweight (BW), height, Body Fat Percentage (BFP), Body Mass Index (BMI), and waist to hip ratio (WHR)) were measured at the Pre 1 and Pre 2. Subjects were then randomly divided into two experimental (Exp; n = 13) and control (C; n = 13) groups. The lottery was used to assign the subjects to the groups. It should be noted that one individual in each group refused to continue the study protocol. Figure 1 shows the consort flow diagram for the study.

### Exhaustive exercise protocol

Based on previous studies, the exhaustive Bruce aerobic test was performed on a treadmill. After 10 min of general warm-up, the subjects started the protocols (10 Min, 4 km/h, 0%). The test consisted of seven 3-min steps, beginning with a slope of 10% and a speed of 1.7 mph and increasing the slope of the device by 2% every 3 min. The speed increased by 1.7 mph until the subjects could no longer continue their activities and expressed complete exhaustion. Finally, 5 min of cooling down at a 4 km/h and a zero slope were performed. The Borg scale of rated perceived exertion (RPE) was utilized during running (3). Both Exp and C groups performed the exercise program twice before starting the Vit D supplementation (or placebo) and after six weeks of intervention.

### Vit D supplementation

**Fig. 2:**
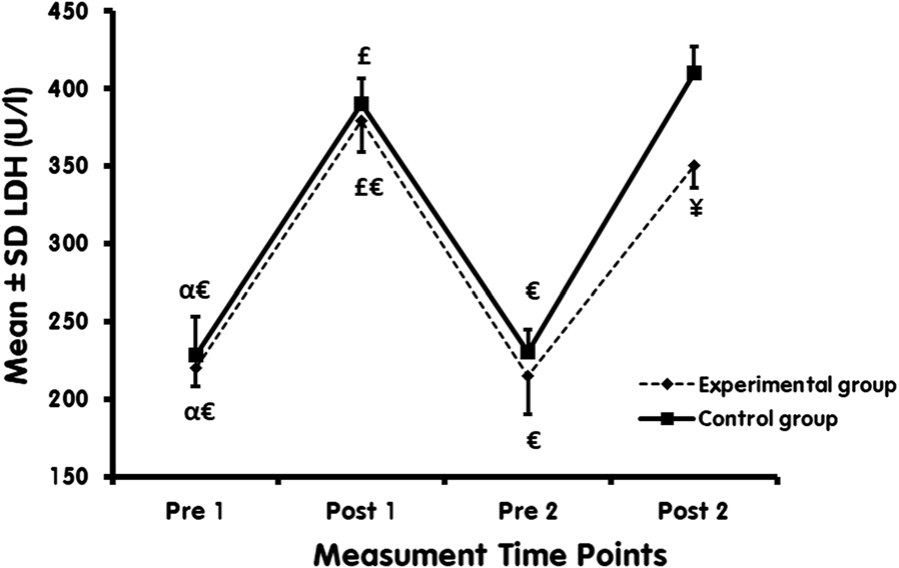
LDH levels at different time points in Exp and C. ^α^: Significantly different compared with post1 within the group. ^£^: Significantly different compared with pre 2 within the group. ^€^: Significantly different compared with post 2 within the group. ¥: Significantly different comparing Exp and C

The Vit D supplementation group received 2000 IU Vit D per day (oral calcifediol; Zahravi Pharmaceutical Company) for six weeks (42 days). The C group also received a placebo (containing paraffin made by Zahravi Pharmaceutical Company, Iran), which was similar to Vit D supplements in shape, color, smell, and taste (Fig. 2).

### Blood sampling

The patient's blood was taken four times; the first and second samples were collected at the start of the study, before (pre1) and immediately after (post1) the initial exercise program. The third and fourth samples were taken after six weeks of intervention, before (pre2) and immediately after (post2) the second exercise protocol. Five ml of blood was taken from the subjects and poured into test tubes and injectors without EDITA for each sampling. The samples were delivered to the lab frozen and centrifuged for 10 min at 3000 rpm, following which the serum was separated and stored at − 80 °C.

Vit D levels were assessed by the direct competitive immunoassay method and the liver enzymes (ALT, AST, GGT, and ALP) with the ELISA method (Greiner Bio-One kit made in Germany). Serum LDH and CK levels were assessed using Pars Azmon kits (Tehran, Iran) with a 4–5 units' sensitivity with Hitachi auto-analyzer (model 902 made in Japan).

### Statistical methods

Descriptive statistics were used to determine the mean and standard deviation of variables, and Shapiro–Wilk test was used to examine the normality of the distribution. Within-group changes were examined using a dependent *t* test and an independent test was used to assess the difference in differences (DID) (Pre 2–Pre 1) in the anthropometric characteristics. While between-group comparisons were made using a repeated-measures ANOVA, Bonferroni's post hoc test. All analyses were performed with SPSS software (version 26) at a significance level of *P* < 0.05.

## Results

The anthropometric characteristics of the subjects are presented in Table 1. Comparing Pre 1 and Pre 2, BW, BMI, BFP, and WHR were significantly decreased in the Exp and even though not statistically significantly increased in the C (except in PFB; *P* = 0.041). The results of DID (Pre 2–Pre 1) show significant differences between Exp and C in BW (*P* = 0.039), BMI (*P* = 0.025), BFP (*P* = 0.043), and WHR (*P* = 0.035).

**Table 1:** Mean ± SD of anthropometric indices before the intervention among the groups

Variables	Exp (n = 12)		C (n = 12)		*P* value^a^
	Pre 1	Pre 2	Pre 1	Pre 2	
Age (years)	24.33 ± 2.7	–	25.83 ± 3.18	–	
Height (cm)	174.72 ± 6.16	–	173.33 ± 5.10	–	
BW (kg)	76.90 ± 11.08	75.96 ± 11.67	77.90 ± 7.13	78.40 ± 6.89	0.039¥
P Value^b^	0.003*		0.056		
BMI (years)	25.80 ± 2.93	25.12 ± 2.79	25.91 ± 1.88	26.08 ± 1.83	0.025¥
P Value^b^	0.003*		0.054		
BFP (%)	27.66 ± 2.80	24.75 ± 2.65	26.01 ± 2.42	26.95 ± 2.84	0.043¥
P Value^b^	0.001*		0.041*		
WHR (cm)	0.82 ± 0.051	0.81 ± 0.032	0.83 ± 0.023	0.83 ± 0.044	0.035¥
P Value^b^	0.047*		0.062		

BW: Bodyweight; BMI: Body Mass Index; BFP: Body Fat Percentage; WHR: Waist–Hip Ratio

*P* values superscript with “a” is calculated using an independent t-test for comparing Δ between groups

*P* values superscript with “b” is calculated using dependent t-test for comparing pre-test and post-test within groups

* Significantly different comparing Pre and Post

The results of this study indicate a significant increase in liver enzymes (ALT, AST, GGT, and ALP) in both C and Exp following the EAE (comparing Pre 1 and Post 1). Also, after six weeks of Vit D supplementation, the liver enzymes increased significantly in both C and Exp following EAE (comparison of Pre 2 and Post 2). In Exp, however, there was a significant decrease in ALT, AST, GGT, and ALP in Post 2 compared to Post 1 (Table 2).

**Table 2:** Liver enzymes and Vit D levels at different time points in experimental and control

Variables		Pre 1	Post 1	Pre 2	Post 2	*P* value^a^
ALT (U/L)	Exp	25.18 ± 2.24^α£€^	36.17 ± 1.28^£€^	19.18 ± 1.43^€^	28.08 ± 2.33	0.001
	C	26.21 ± 1.19^α€^	34.45 ± 1.39^£^	27.29 ± 1.26^€^	36.18 ± 1.67	0.001
*P* value^b^		0.298	0.093	0.001	0.001	
AST (U/L)	Exp	21.08 ± 1.32^α£€^	30.06 ± 1.16^£€^	16.15 ± 1.44^€^	24.13 ± 1.35	0.001
	C	23.04 ± 1.27^α€^	31.23 ± 2.21^£€^	24.21 ± 1.64^€^	33.14 ± 1.36	0.001
*P* value^b^		0.247	0.264	0.011	0.001	
GGT (U/L)	Exp	23.06 ± 1.33^α£€^	34.11 ± 1.40^£€^	19.20 ± 1.27^€^	27.07 ± 1.37	0.001
	C	24.15 ± 1.23^α€^	35.09 ± 1.14^£€^	24.01 ± 1.60^€^	35.21 ± 2.06	0.001
*P* value^b^		0.139	0.204	0.019	0.001	
ALP (U/L)	Exp	180.09 ± 2.24^α£€^	178.11 ± 1.35^£€^	178.21 ± 1.39^€^	192.21 ± 1.23	0.001
	C	183.15 ± 1.28^α€^	204.27 ± 1.43^£€^	184.34 ± 1.22^€^	209.61 ± 1.02	0.001
*P* value ^b^		0.111	0.909	0.001	0.001	
Vit D (ng/mL)	Exp	22.13 ± 2.15^α£€^	25.19 ± 1.45^£€^	45.34 ± 1.24^€^	49.13 ± 1.20	0.001
	C	24.15 ± 1.51^α€^	26.18 ± 2.43^£€^	23.08 ± 1.11^€^	28.32 ± 1.07	0.001
*P* value^b^		0.068	0.164	0.001	0.001	

AST: Aspartate Aminotransferase; ALT: Alanine Aminotransferase; GGT: Gamma-Glutamyl Transferase; ALP: Alkaline Phosphatase; Vit D; Vitamin D

*P* values superscript with “a” is calculated using repeated measures ANOVA test for comparing different time points; *P* values superscript with “b” is calculated using independent *t* test for comparing between groups at each time points

^α^Significantly different compared with post1 within the group

^£^Significantly different compared with pre 2 within the group

^€^Significantly different compared with post 2 within the group

The results of the independent *t* test showed significantly lower ALT (*P* = 0.001; *P* = 0.001), AST (*P* = 0.011; *P* = 0.001), GGT (*P* = 0.018; *P* = 0.001), and ALP (*P* = 0.001; *P* = 0.001) in the Exp in Pre 2 and Post 2 compared with C. While no such differences were observed between Exp and C in the variables above in Pre 1 and Post 1 (Table 2).

Table 2 shows a significant increase in Vit D in both C and Exp following the EAE (comparing Pre 1 and Post 1). After six weeks, Vit D levels increased significantly in Exp, while a significant decrease was observed in C (comparison of Pre 1 and Pre 2). Also, Vit D increased significantly in both C and Exp following the second EAE session (comparison of Pre 2 and Post 2). However, the increase in Vit D in Post 2 was significantly higher than in Post 1 in Exp. The independent t-test showed significantly higher Vit D in the Exp in Pre 2 (*P* = 0.001) and Post 2 (*P* = 0.001) compared with C.

The present study results indicate a significant increase in muscle damage indices (LDH and CK) in both C and Exp following the EAE (comparing Pre 1 and Post 1). Also, after six weeks of Vit D supplementation, the muscle damages indices (LDH and CK) increased significantly in both C and Exp following EAE (comparison of Pre 2 and Post 2). However, there was a significant decrease in LDH, CK in Post 2 compared to Post 1 in Exp.

**Fig. 3:**
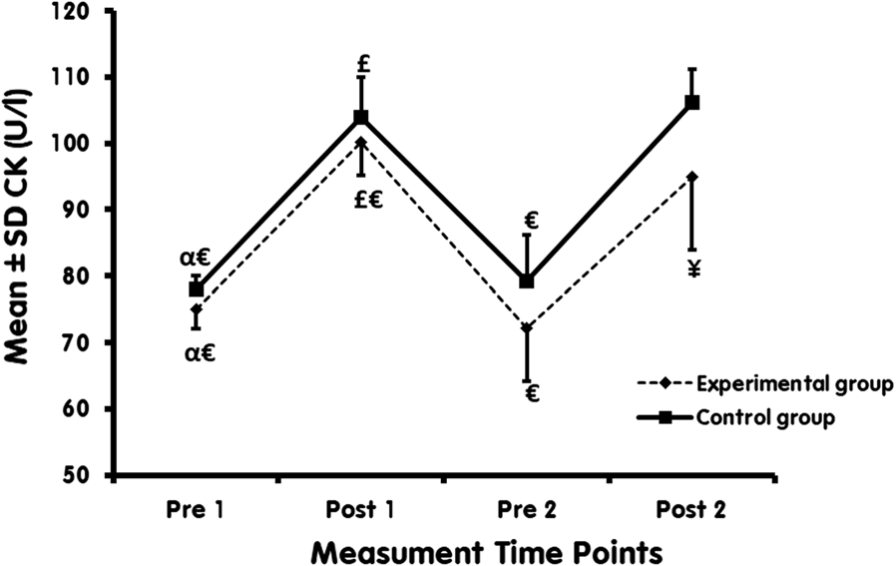
CK levels at different time points in Exp and C. ^α^: Significantly different compared with post1 within the group. ^£^: Significantly different compared with pre 2 within the group. ^€^: Significantly different compared with post 2 within the group. ¥: Significantly different comparing Exp and C

The independent t-test showed significantly lower LDH, CK in the Exp compared with C in Post 2 (*P* = 0.001). While no such differences were observed between Exp and C in the LDH, CK in Pre 1 (*P* = 0.325), Post 1 (*P* = 0.431), and Pre 2 (*P* = 0.067) (Figs. 2, 3).

## Discussion

This study results showed that although single bouts of EAE lead to an increase in liver enzymes, short-term vitamin D supplementation downregulates the liver enzymes production following EAE. The EAE has been shown to increase electron penetration, mitochondrial oxygen consumption, and the production of free radicals, leading to fat peroxidation, dysfunction of membrane enzymes, and subsequent cell membrane degradation. Therefore, an increase in liver enzymes can indicate cell leakage and structural cell damage. There was also a significant increase in vitamin D levels following EAE. Considering the phospholipid bilayer structure of cell membrane and vitamin D as a fat-soluble vitamin, increased vitamin D releasement into the blood following membrane degradation might be one of the possible mechanisms. Interestingly, the present study results showed that short-term vitamin D supplementation significantly downregulates liver enzymes following EAE. The exact mechanism of the protective effects of vitamin D on the downregulation of liver enzymes following EAE is not yet fully understood. Generally, short-term vitamin D supplementation is likely to reduce insulin resistance via lowering insulin and glucose levels [19]. It also prevents hepatic fat accumulation by oxidation of fats, inhibiting lipogenesis, and regulating the circulating fatty acids [20].

The present study's findings indicated significant increments in skeletal muscle cell damage indicators (CK and LDH) in Exp and C at Post1. Comparing Post2 with Post1, the skeletal muscle cells damage indicators decreased significantly in Exp. However, no significant change was observed in C. Consistent with these results, Tofighi et al., (2015) observed increased skeletal muscle cells damage indicators immediately, 24, and 48 h after EAE in all Groups (30 men, divided into three groups: control, BCAA, and CARBS) [8]. In a study on 20 inactive healthy women, Ajam Zibad et al., (2016) reported that a single bout of intense acute resistance exercise significantly increased serum levels of LDH, CK TNF-α, and MDA [9]. EAE -induced muscle damage increases the inflammatory mediators and cytokines levels leading to the increased circulating neutrophil, cell death, and phagocytic clearance in skeletal muscle [21]. As a result, cell membranes disintegrate, allowing muscle proteins like LDH and CK to leak into the intercellular fluid and eventually into the bloodstream[22]. EAE-induced fatigue also increases cell membrane permeability to intracellular free calcium ions in muscle fibers, which could result in sodium–potassium pump dysfunction, cell membrane instability, and activation of proteases (elastases and myeloperoxidases) and intracellular lipases (phospholipases) [21, 22]. Over-regulation of proinflammatory cytokines (e.g., IL-6 and TNF-), increased NF-JB activation, and E3 ubiquitin-ligases MuRF-1 expression are all involved in EAE-induced protein degradation and muscle damage [23], which ultimately leads to increased circulating CK and LDH in response to myofibrillar degradation [24].

As previously stated, skeletal muscle cell damage indicators increased in Exp at both Post1 and Post 2 following EAE. Still, this increase was significantly reduced in Post2 compared to Post1, suggesting that vitamin D supplementation may effectively reduce EAE-induced skeletal muscle cell damage. Consistent with our results, Pilch et al., [23] and Choi et al., [24] reported that vitamin D improves EAE-induced skeletal muscle cell damage. Vit D modulates several functions of skeletal muscles via increasing the expression of myogenic factors in satellite cells, enhancing muscle differentiation, growth, and regeneration, modulating myostatin via increased follistatin expression, modulating phosphate metabolism by affecting mitochondrial health via modulating calcium metabolism, thus reducing the muscle injury and increasing the recovery from EAE sessions [17].

In the present study, increased serum levels of vitamin D might be a possible reason for reducing skeletal muscle cell damage indicators following EAE in the Exp due to the protective role of vitamin D against cell damage [25]. Stojanovic et al., (2021) reported that increasing vitamin D levels in individuals with vitamin D deficiency reduces EAE-induced muscle damage [26]; Vitamin D receptors are likely to cause this by their genomic effects on skeletal muscle [27]. Also, vitamin D plays an important role in reducing EAE-induced skeletal muscle cell damage and inflammation by regulating immune system function (such as macrophages and T cells) [24] and modulating MAPK and NF-KB activation [28].

### Strengths and limitations

The strengths of the present study included focusing on the novel questions using a randomized, single-blind, placebo-controlled trial with a low dropout rate. The limitation of the present study was the small sample size due to the COVID-19 and no financial support that obliged us not to measure some of the blood indicators and related gene expression, which can be a subject for further research.

## Conclusion

A single bout of EAE-increased the hepatic and skeletal muscle cells damage indicators, while short-term vitamin D supplementation decreased the increment of hepatic and skeletal muscle cells damage indicators following EAE. Therefore, vitamin D supplementation is recommended to overweight, untrained individuals to reduce the EAE-induced increment of liver and skeletal muscle cell damage indicators. Further studies are needed to find the most effective supplementation dose and duration.

## Data Availability

The datasets used and/or analyzed during the current study are available from the corresponding author on reasonable request.

## Abbreviations

BW: Bodyweight
BMI: Body Mass Index
BFP: Body Fat Percentage
WHR: Waist–hip ratio
Vit D: Vitamin D
CK: Creatine kinase
LDH: Lactate dehydrogenase
AST: Aspartate aminotransferase
ALT: Alanine aminotransferase
GGT: Gamma-glutamyl transferase
ALP: Alkaline phosphatase
